# Supplementary material for: Revealing time’s secrets at the National Theatre of Costa Rica via innovative software for cultural heritage research
Source: Sci Rep. 2021 Apr 20;11:8560. doi: 10.1038/s41598-021-88066-1 (PMC8058347; doi:10.1038/s41598-021-88066-1)
Supplement: Supplementary file 1 — Supplementary Informations. [file 41598_2021_88066_MOESM1_ESM.pdf]

Revealing time's secrets at the National Theater of Costa Rica via innovative software for cultural heritage research

M.D. Barrantes-Madrigal[1,2], T. Zúñiga-Salas[3], R.E. Arce-Tucker[4], A. Chavarría-Sibaja[2,5], J. Sánchez-Solís[6], J. Mena-Vega[5], K. Acuña-Umaña[2,5], M. Gómez-Tencio[1], K. Wang-Qiu[3], F. Lizano-Sánchez[1], C. Marín-Cruz[7], and O.A. Herrera-Sancho[2,5,8,9,*]

[1]Escuela de Química, Universidad de Costa Rica, 2060 San Pedro, San José, Costa Rica
[2]Centro de Investigación en Ciencias Atómicas Nucleares y Moleculares, Universidad de Costa Rica, 2060 San Pedro, San José, Costa Rica
[3]Escuela de Artes Plásticas, Universidad de Costa Rica, 2060 San Pedro, San José, Costa Rica
[4]Facultad de Microbiología, Universidad de Costa Rica, 2060 San Pedro, San José, Costa Rica
[5]Escuela de Física, Universidad de Costa Rica, 2060 San Pedro, San José, Costa Rica
[6]Escuela de Ingeniería Eléctrica, Universidad de Costa Rica, 2060 San Pedro, San José, Costa Rica
[7]Teatro Nacional de Costa Rica, 5015-1000 San José, Costa Rica
[8]Centro de Investigación en Ciencia e Ingeniería de Materiales, Universidad de Costa Rica, 2060 San Pedro, San José, Costa Rica
[9]Instituto de Investigaciones en Arte, Universidad de Costa Rica, 2060 San Pedro, San José, Costa Rica
[*]oscar.herrerasancho@ucr.ac.cr

Supplementary Table S1. Time required per method in a multi-analytical study. Distribution of time on-site and off-site the NTCR required for each method carried out in this study. In general, the distribution of time required for each step of the on-site and off-site analysis may vary. The time spent will depend on the number of samplings carried out with our software, the state of conservation of the artwork, and its size. In this estimation, we consider that 1 day corresponds to 8 hours and 1 month corresponds to 25 days.

Method	Description	Time on-site	Time off-site
Multispectral Imaging	Photography acquisition and processing	3 days	15 days
Software development	Software development<br>Analysis of luminosity with the *RegionOfInterest* program<br>Measure of crystals diameters with the *CrystalDistribution* program	–<br>–<br>–	3 months<br>5 days<br>5 days
Microscopy and spectroscopy analysis	Painting sampling and processing<br>Optical microscopy<br>SEM – EDX<br>FTIR-ATR	7 days<br>–<br>–<br>–	21 days<br>1 month<br>2 days<br>1 days
Analysis of environmental and biological factors	Microorganisms analysis<br>Environmental analysis	1 day<br>21 days	3 months<br>3 months

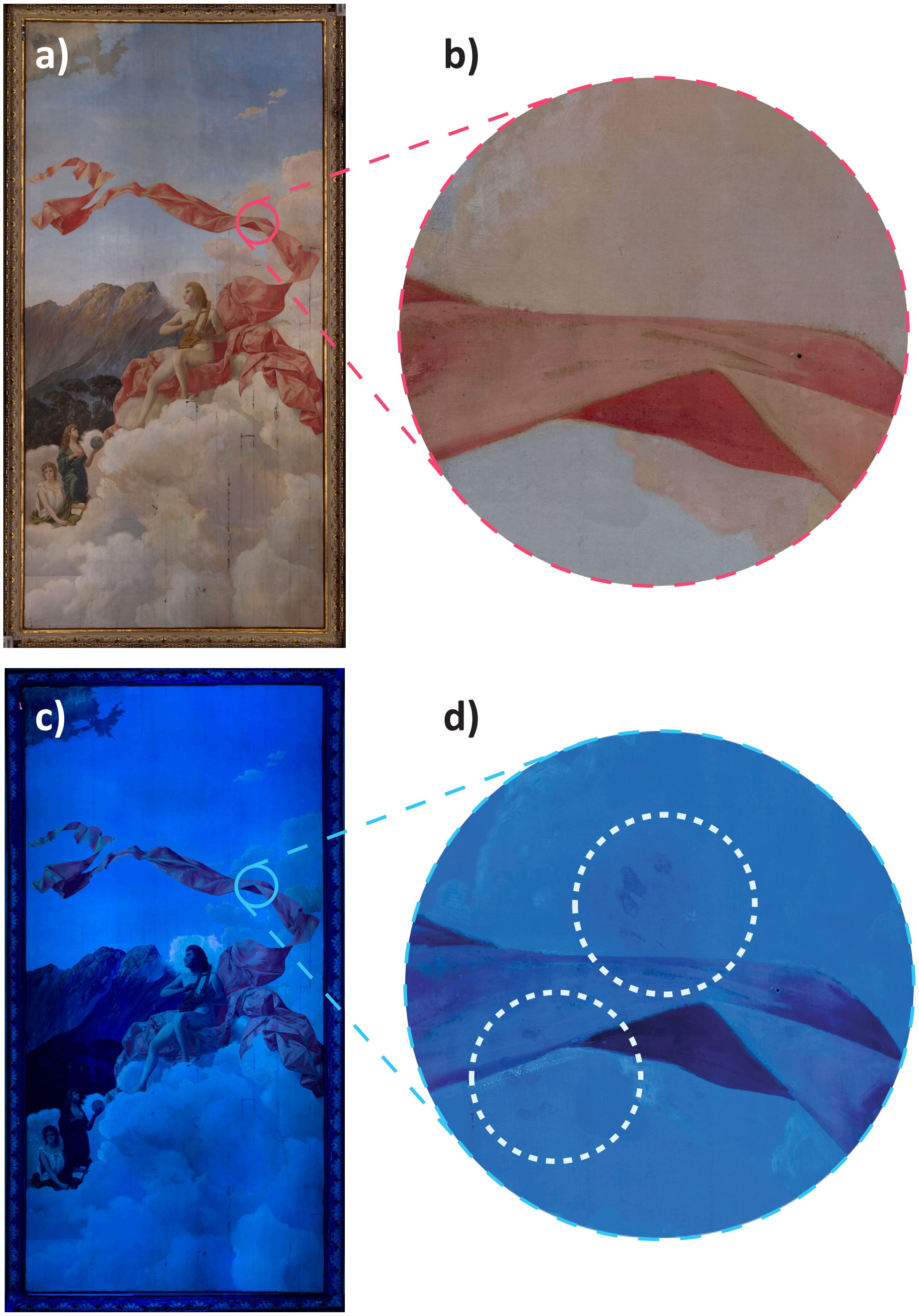

Supplementary Figure S1. Our impact on paintings: Human fingerprints damage. Visible photograph of **(a)** *Musas I* presenting precisely the working grids 29 and 35 **(b)**, the working grid is displayed in Fig. 2f (top). **(c)** Ultraviolet fluorescence (UVF) multispectral image (MSI) showing human fingerprints damage caused at the surface of the studied artwork **(d)**, see categorised damage number 6 in Fig. 7b.4. Panel b) shows the need of using MSI since these damages are not visible. These fingerprints were caused possibly by a person who leaned on the paint during a cleaning or restoration process.
